# Supplementary material for: Pattern generation and symbolic dynamics in a nanocontact vortex oscillator
Source: Nat Commun. 2020 Jan 30;11:601. doi: 10.1038/s41467-020-14328-7 (PMC6992810; doi:10.1038/s41467-020-14328-7)
Supplement: Supplementary file 1 — Supplementary Information [file 41467_2020_14328_MOESM1_ESM.pdf]

Supplementary Information

“Pattern generation and symbolic dynamics in a nanocontact vortex oscillator”

Yoo *et al.*

SUPPLEMENTARY FIGURES

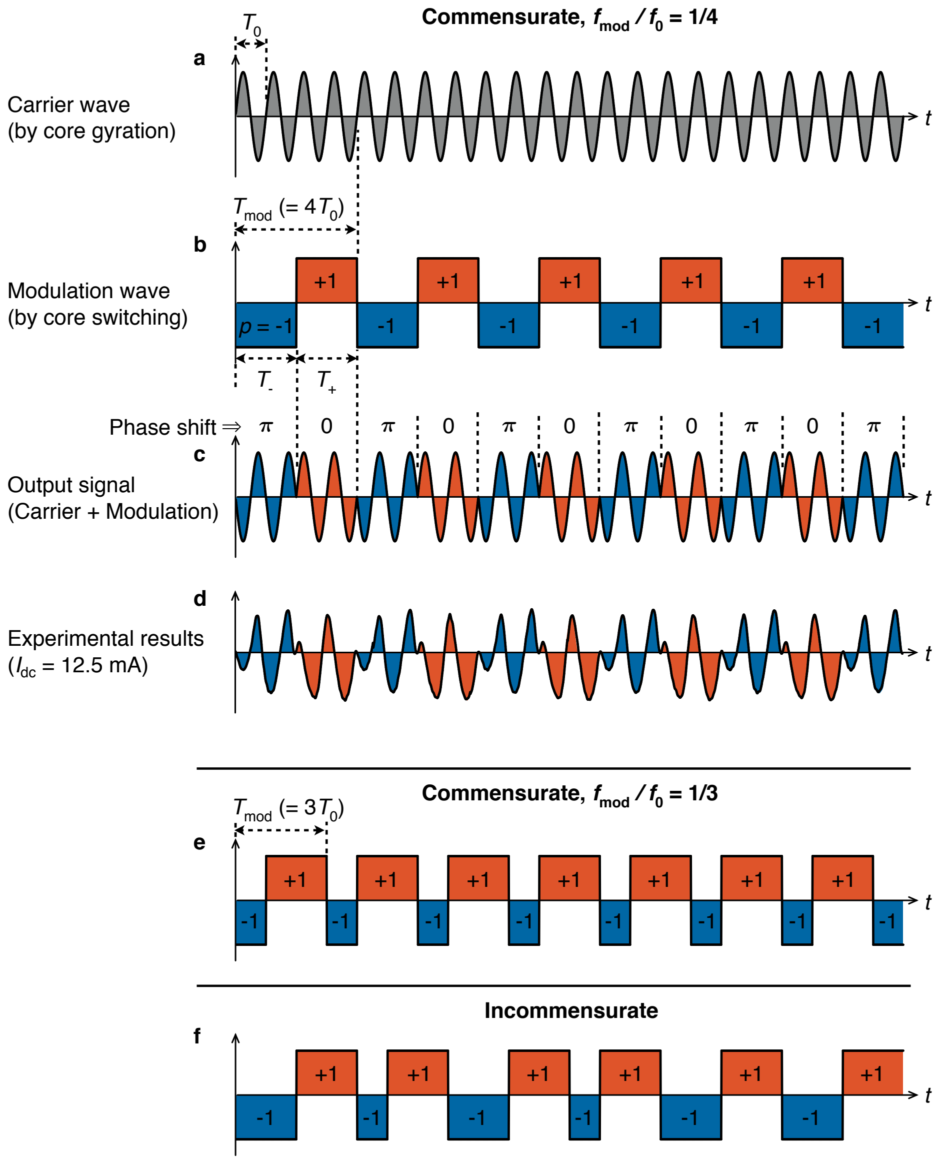

Supplementary Figure 1 | Ratio between fundamental and modulation frequencies. **a**, A sinusoidal carrier wave with a period of $T_0 = 1/f_0$. **b**, A square modulation wave for the case of $f_{mod}/f_0 = 1/4$. T_+ and T_- are duration times for $p = +1$ and -1, respectively.The red and blue colours represent the core polarity, $p = +1$ and -1, respectively. **c**, The output signal obtained from the carrier and modulation by a phase shift. The shifted phase are represented above the graph. The red and blue colours correspond to those in b. **d**, An experimentally measured time-resolved voltage oscillation at the commensurate state ($f_{mod}/f_0 = 1/4$). **e**, as in b but in the case of $f_{mod}/f_0 = 1/3$. **f**, as in b but in the incommensurate case.

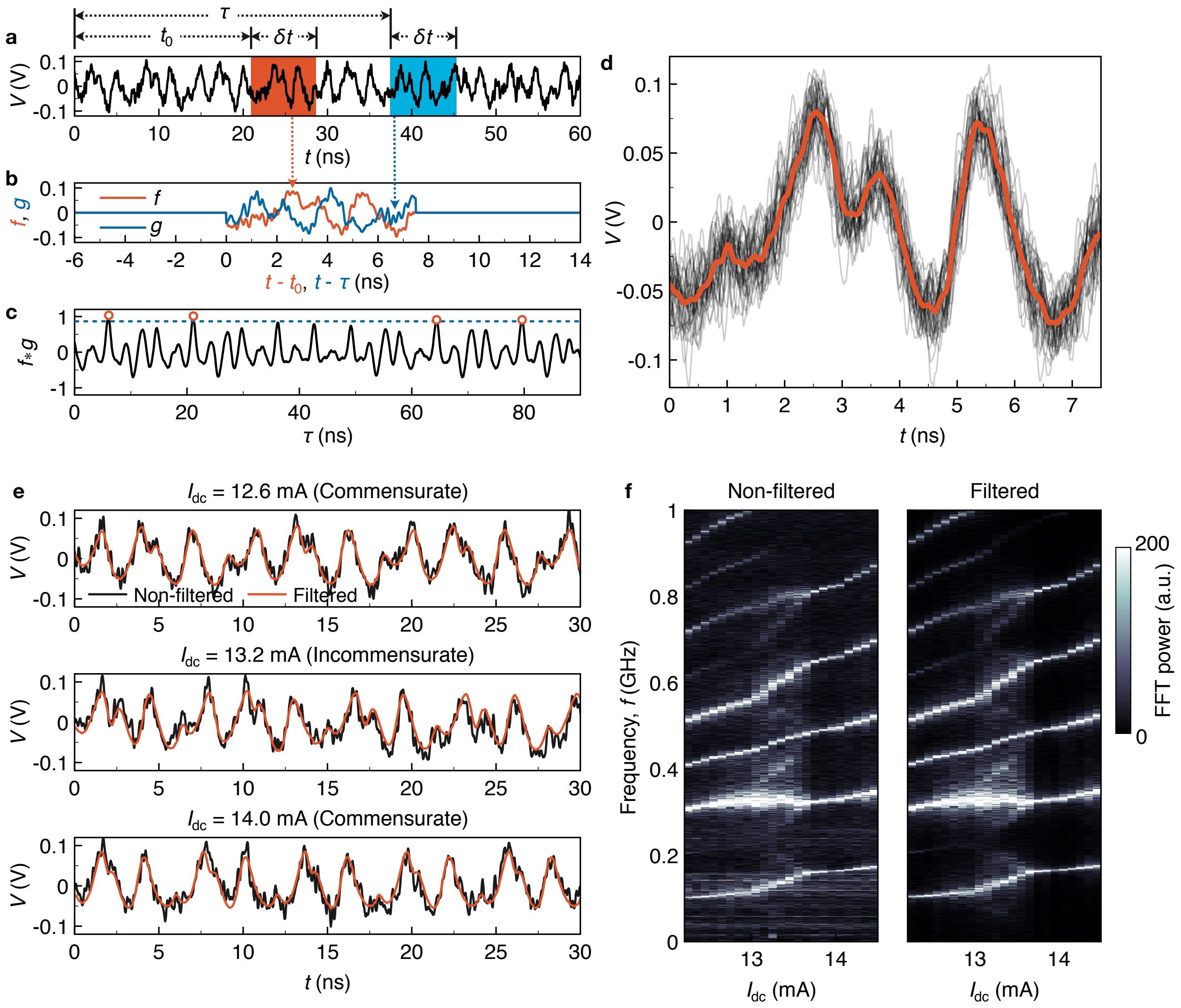

Supplementary Figure 2 | Noise reduction filter. **a**, A measured non-filtered time trace at $I_{\mathrm{dc}} = 13.2$ mA. The red and blue areas represent $t_0 \leq t < t_0 + \delta t$ and $\tau \leq t < \tau + \delta t$ which are the regions of the functions of f and g, respectively. **b**, $f(t - t_0)$ (red line) and $g(t - \tau)$ (blue line) which are correspond with the colour regions in a. **c**, $(f(t - t_0) * g(t - \tau))$ as a function of τ. The dashed line represents a tolerance value and red circles indicate the valid peak positions ($\tau_i = \tau_1, \tau_2, \cdots$) which heights are larger than the tolerance. **d**, Collected short-term waveforms from τ_i, $V(\tau_i \leq t \leq \tau_i + \delta t)$ (black lines). The red curve is a filtered data obtained by averaging the black lines. **e**, Measured non-filtered signals (black) and filtered signals (red). **f**, Power of the fast Fourier transformation (FFT) as a function of the input current, I_{dc}, calculated from the non-filtered (left) and filtered (right) time traces.

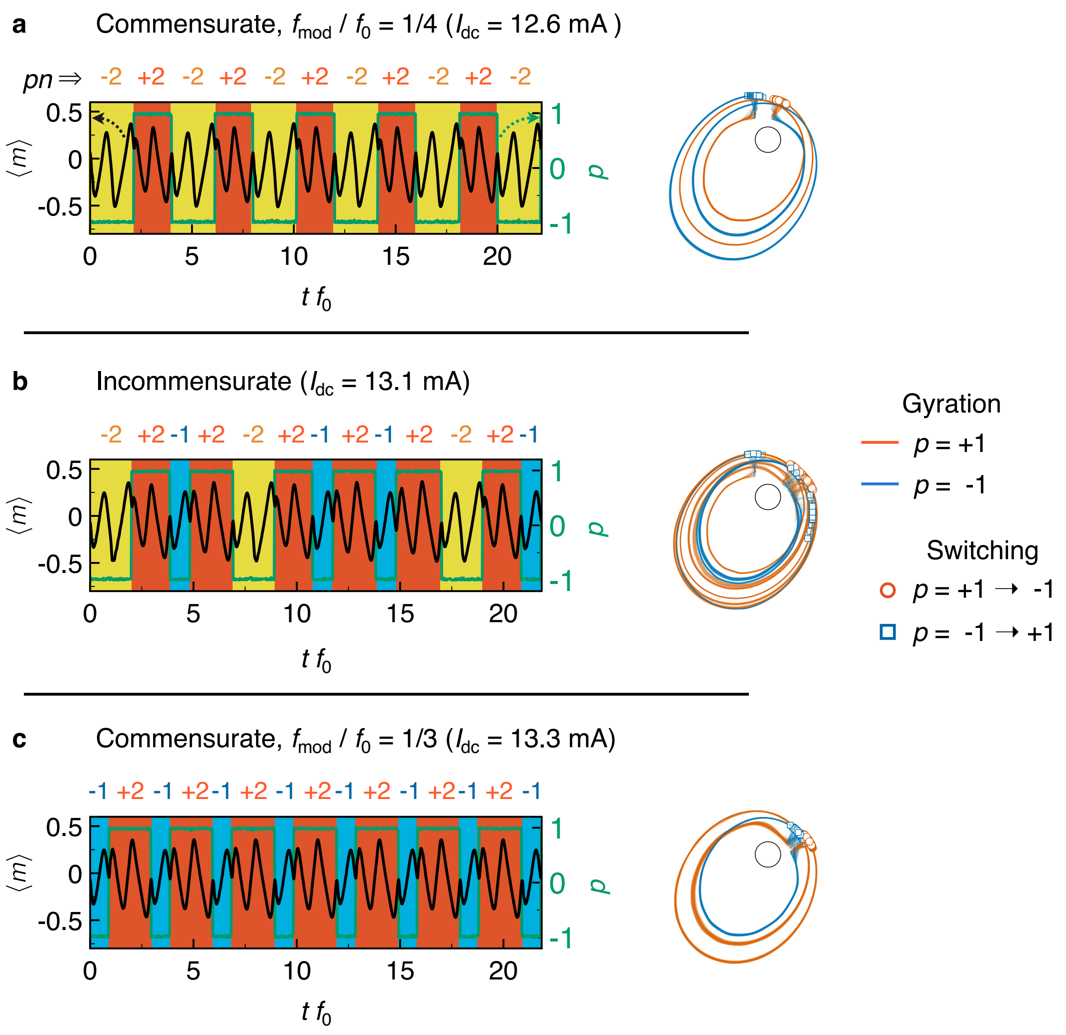

Supplementary Figure 3 | Pattern generations from micromagnetic simulations a, Time evolution of an average of magnetization, $\langle m \rangle$ for the commensurate ($f_{\text{mod}}/f_0 = 1/4$) (black line). x axis is normalised by $1/f_0$, where f_0 is the gyration frequency. The green line is the core polarisation associated with the right axes. The colour regions indicate corresponding *pn* patterns defined in Fig.2g in the main text. On the right side, the corresponding core trajectories are presented. The red and blue lines are the core trajectories when $p = +1$ and -1, respectively. The red circles and blue squares indicate the core switching positions from $p = +1$ to -1 and from -1 to $+1$, respectively. **b**, as in a but in the incommensurate case. **c**, as in a but in the case of $f_{\text{mod}}/f_0 = 1/3$.

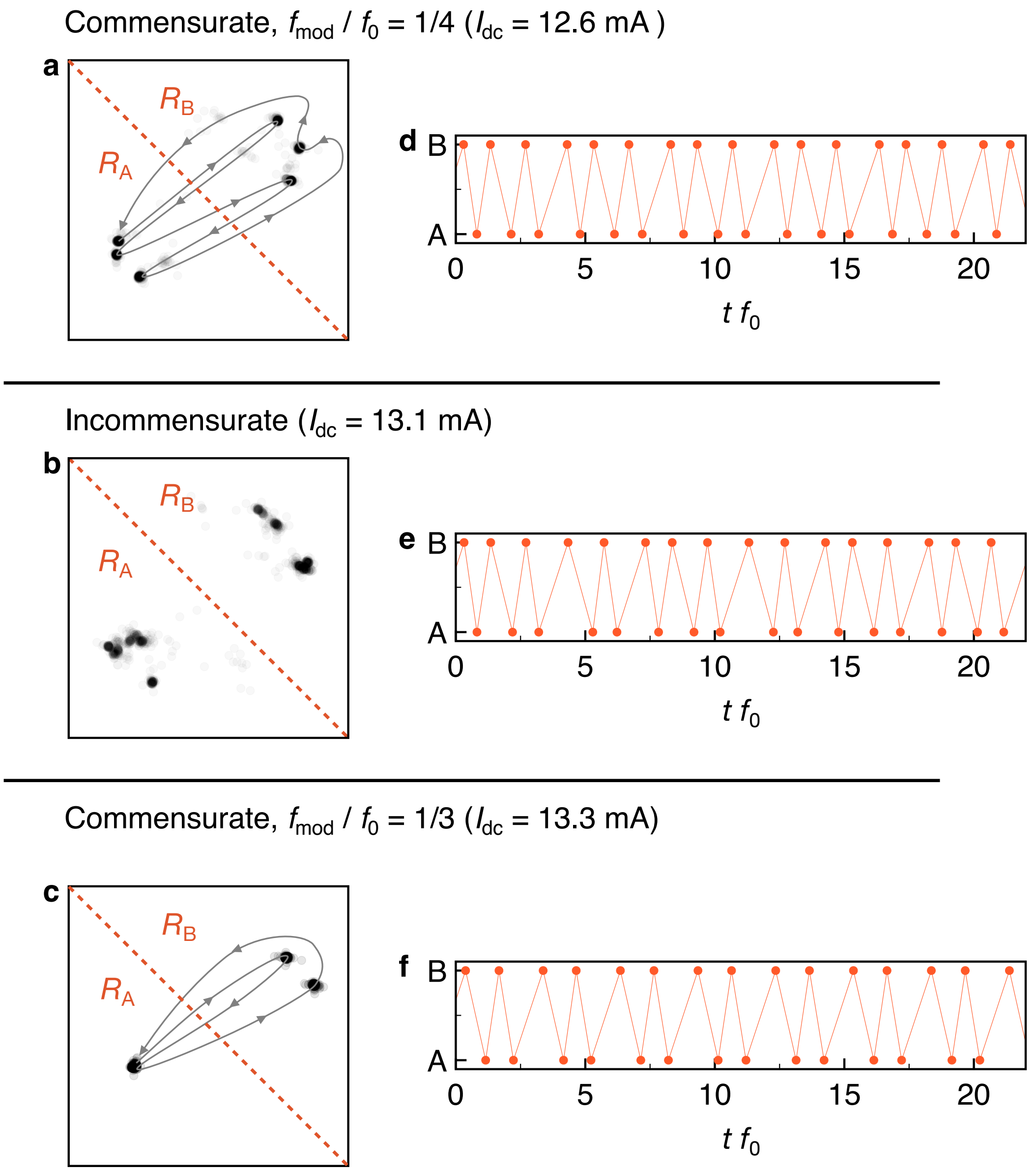

Supplementary Figure 4 | Symbolic dynamics using a different partition. a-c, Poincaré maps for I_{dc} = 12.6 mA, 13.2 mA, and 14.0 mA. The red dashed line is a new partition to divide the regions, R_A and R_B for symbolic dynamics. The gray arrows in a and c show a dynamics of the intersection points in the case of the commensurate state. **d-f**, Symbolic dynamics based on the newly defined partition.

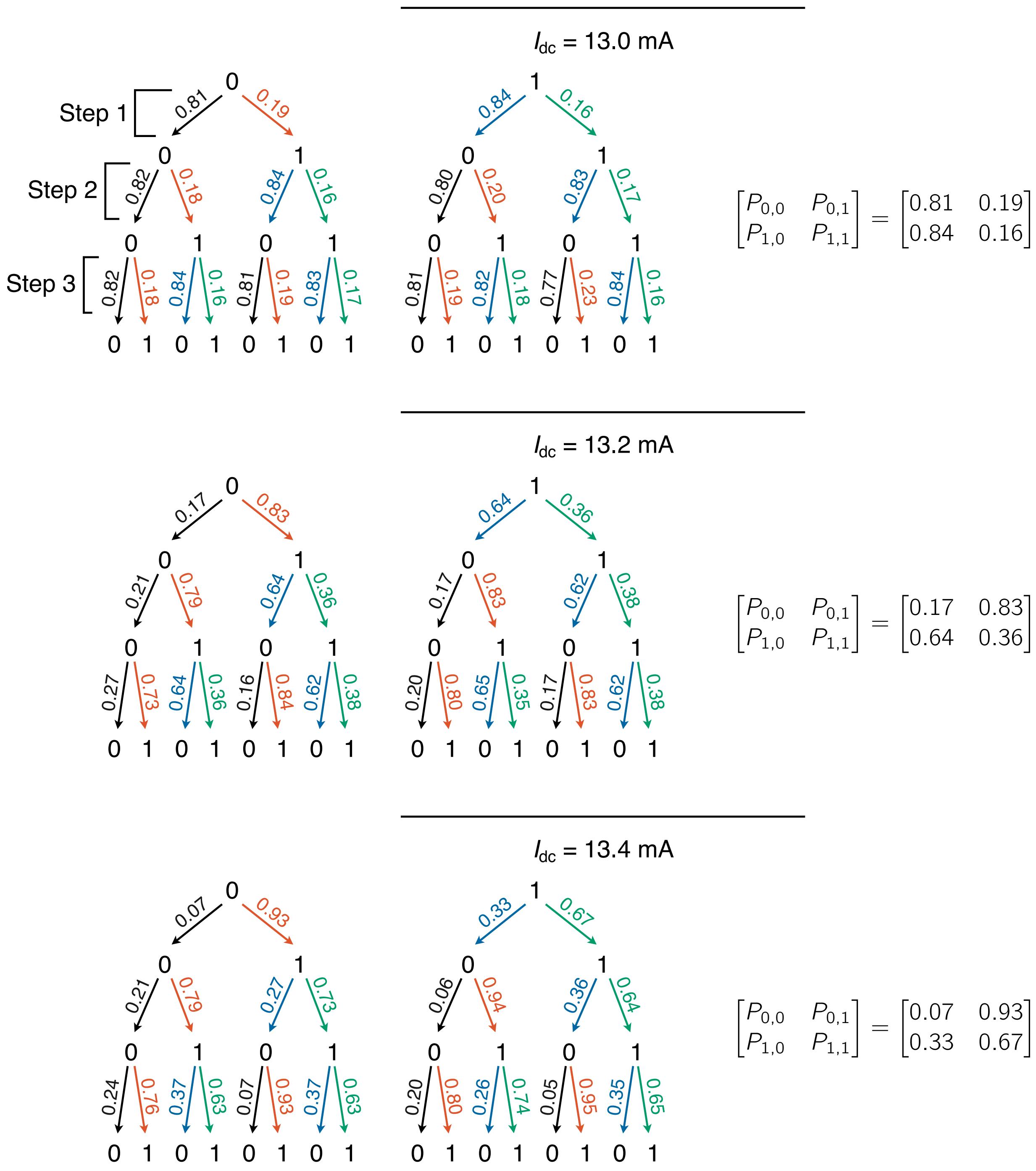

Supplementary Figure 5 | Tree diagrams and Markov matrices. (Left) Tree diagrams of moving for three steps. Numbers at the arrows show probabilities of the moving including their previous states. The black, red, blue, and green colours represent the movings of 0 → 0, 0 → 1, 1 → 0, and 1 → 1, respectively. (Right) Markov matrices for each I_{dc} by assuming one step memory dynamics. $P_{i,j}$ indicates the probability of the moving from i to j. Source data are provided as a Source Data file.

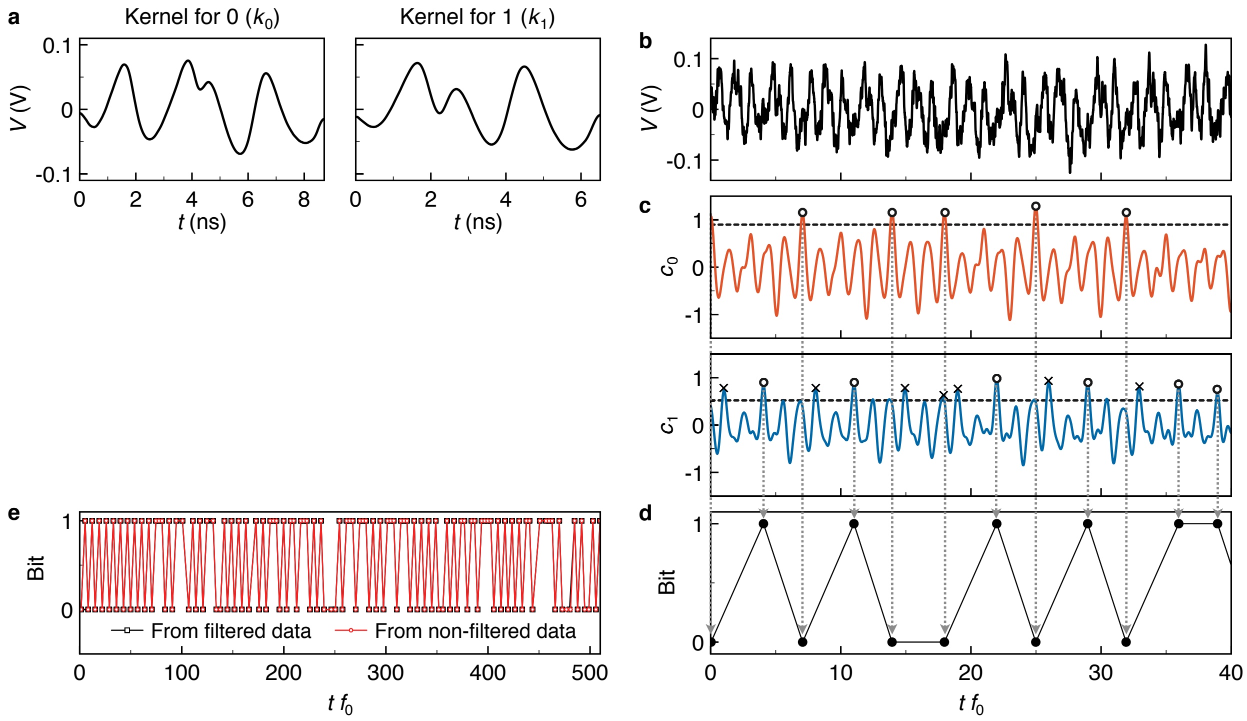

Supplementary Figure 6 | Bit sequences from non-filtered data by convolutions. **a**, Kernel functions of “0” and “1” for I_{dc} = 13.2 mA. **b**, A non-filtered time trace at I_{dc} = 13.2 mA. **c**, Convolutions, c_0 and c_1, between the non-filtered time trace and the kernels, k_0 (Top) and k_1 (Bottom), respectively. The dashed lines show prescribed tolerances for c_0 and c_1. The open circles are valid peaks for embedding bits. Cross marks in c_1 are fallacious peaks which need to be ignored . **d**, An obtained bit sequence recognised from the non-filtered data by the convolutions. The gray dashed arrows from c show the corresponding peaks. **e**, Red circles indicate bit sequences directly obtained from the non-filtered data, and black squares represent the bits calculated from the filtered data.

SUPPLEMENTARY NOTES

Supplementary Note 1. Ratio between fundamental and modulation frequencies

In this note we discuss the meaning of the frequency ratio, f_{mod}/f_0, as explained in the main text (Fig. 1c), where f_0 and f_{mod} are fundamental and modulation frequencies, respectively. The oscillatory signals from the nanocontact vortex oscillator (NCVO) can be considered as phase-modulated sinusoidal waves by the vortex core switching, because the core reversal inverts a sense of the core gyration. The sinusoidal oscillations of the carrier wave originates from the vortex core gyration (Supplementary Figure 1a), whose frequency corresponds with the gyration frequency, $f_0 = 1/T_0$, where T_0 is a time period of the gyration; the core switching generates the a square-shaped phase modulation signal of which frequency is f_{mod} =

$1/(T_{\mathrm{mod}}) = 1/(T_+ + T_-)$, where T_{mod} is a period of the modulation and T_+ and T_- are dwell times for the upward core ($p = +1$) and downward core ($p = -1$), respectively. p is a core polarity. An example of the modulation of $f_{\mathrm{mod}} = f_0/4$ and the modulated output signal are represented in Supplementary Figure 1b and c, respectively. The schematic output signal is qualitatively in a good agreement with the experimental results (compare with Supplementary Figure 1d).

From Supplementary Figure 1a-c, we can conclude that f_{mod}/f_0 can be expressed approximately as

$$\frac{f_{\mathrm{mod}}}{f_0} = \frac{T_0}{T_{\mathrm{mod}}} = \frac{1}{n_+ + n_-}, \tag{S1}$$

in the typical commensurate state, where $n_+ = T_+ f_0$ and $n_- = T_- f_0$ are required gyration numbers for the core switching in the cases of $p = +1$ and -1, respectively. In Supplementary Figure 1b, for example, $n_+ = 2$ and $n_- = 2$, thus $f_{\mathrm{mod}}/f_0 = 1/4$. In Supplementary Figure 1e, $n_+ = 2$ and $n_- = 1$, thus $f_{\mathrm{mod}}/f_0 = 1/3$. For the incommensurate case, we replace the gyration numbers by their time-averaged values, $\langle n_+ \rangle$ and $\langle n_- \rangle$, respectively.

Supplementary Note 2. Noise reduction filtering

To reduce thermal noise from the measured time traces, we refine them by averaging short-period waveforms. This method is available in this case, since the output signals are composed by only few simple patterns even in the incommensurate state (Supplementary Figure 3), therefore, we can find sufficient numbers of similar short-term waveforms from long-term measured data. The process are stated below.

1. $V(t)$ is the measured time-domain signal. Choose an interesting short-time period ($t_0 \le t < t_0 + \delta t$), then obtain the kernel function, $f(t - t_0) = V(t - t_0)\chi(t)$, where $\chi(t)$ is a step function defined as

$$\chi(t) = \begin{cases} 1 & \text{if } 0 \le t < \delta t, \\ 0 & \text{if otherwise.} \end{cases} \tag{S2}$$

   In this study, $\delta t = 7.5$ ns (red region in Supplementary Figure 2a and red curve in Supplementary Figure 2b).

2. Set $g(t - \tau) = V(t - \tau)\chi(t)$ (blue region in Supplementary Figure 2a and blue curve in Supplementary Figure 2b).

3. Calculate the convolution $f(t - t_0) * g(t - \tau)$ as a function of τ (Supplementary Figure 2c).

4. Find positions of peaks, τ_i, whose heights are larger than a predetermined tolerance value (Supplementary Figure 2c).

5. Collect the waveforms from $V(\tau_i \leq t < \tau_i + \delta t)$ (black curves in Supplementary Figure 2d).

6. Average all the collected short-term traces (red curve in Supplementary Figure 2d).

7. Repeat the same process for different t_0.

In both the time and frequency domains, we compare the filtered signal with the non-filtered data (Supplementary Figure 2e and 2f), which show reasonably similar results, although some high-frequency information can be lost by the filtering through the averaging process.

Supplementary Note 3. Chaotic pattern generation using micromagnetic simulations

In the main text (Fig. 2a-c), we show chaotic sequences of patterns, pn, from the experiments, where p and n are a core polarity and a required gyration number for the core switching, respectively. Here we show identical pattern generations using micromagnetic simulations at zero temperature.

For the micromagnetic simulations, we solve the Landau-Lifshitz-Gilbert equation with in-plane spin-torque terms using the MuMax3 software [1]. We chose a $1280 \times 1280 \times 20$ nm^3 film which is uniformly discretized with $512 \times 512 \times 1$ finite difference cells. The magnetic parameters used here correspond to those of a NiFe film: an exchange stiffness constant of $A_{\mathrm{ex}} = 10$ pJ m^{-1}, a saturation magnetization of $M_{\mathrm{s}} = 0.8$ MA m^{-1}, a Gilbert damping constant of $\alpha = 0.013$, and a spin polarisation of $P = 0.5$. The spatial distributions of the current and its associated Oersted field were computed using a finite-element method [2]. The initial state used for the simulations is obtained by sweeping an in-plain magnetic field with an applied DC current of 10 mA. To mimic an asymmetry between the $p = +1$ and -1, we apply both a perpendicular DC field of $B_z = -18$ mT and an in-plane DC field of $B_y = 3$ mT simultaneously. In the experiments, the asymmetry is induced by an exchange interaction between the vortex and its magnetic configurations around the vortex.

The simulations were conducted for the commensurate state (Supplementary Figure 3a and 3c) and the incommensurate state (Supplementary Figure 3b), then we extracted a spatial average of magnetization, $\langle m \rangle$, in the saturated magnetisation direction of the reference layer, which is proportional to the output voltage amplitude. We assume that the magnetisation of the reference layer is saturated to $-135°$ from the x axis, and calculated $\langle m \rangle$ only in 100 nm from the center of the nanocontact.

The results are plotted in Supplementary Figure 3 for the commensurate and incommensurate states as well as the corresponding core trajectories. The obtained $\langle m \rangle$ are qualitatively in good agreements with the experimental results (see Fig. 2a-c in the main text); they show sinusoidal oscillations delimited by cusp

points. We also plot the time evolution of the core polarity, p (green lines in Supplementary Figure 3), which directly shows that the cusp positions correspond with the core reversal as discussed in the main text.

In the commensurate cases (Supplementary Figure 3a and 3c), we can find periodic repetition of two patterns of [−2, +2] and [−1, +2], respectively. On the other hand, in the incommensurate state (Supplementary Figure 3b), the sequence is composed of three patterns, [−2, −1, +2], and they are erratically ordered. We also calculated the correlation dimension, D_c, from the time traces of the simulations. D_c is very close to 1 in the commensurate cases ($D_c = 1.05 \pm 0.01$ for $I_{dc} = 12.6$ mA and $I_{dc} = 1.08 \pm 0.002$ for 13.3 mA), while the dimension has a higher value, $D_c = 1.42 \pm 0.02$, in the incommensurate state ($I_{dc} = 13.1$ mA). This result is in good qualitative agreement with the experimental result in Figs. 1 and 2 in the main text, and shows that the chaotic signals at the incommensurate state have deterministic characteristics, although thermal noise affects the dynamics in real experiments.

Supplementary Note 4. Partition on Poincaré map for symbolic dynamics

For symbolic dynamics, we arbitrarily choose a proper Poincaré surface of section and partition. The surface and partition in the main text are one of a successful case for generating meaningful bit sequences. Here, by using an unsuitable partition on the same surface, we show an example of failing to generate bit sequences. In Supplementary Figure 4a-c, we display the Poincaré maps on the Poincaré section and their dynamics using gray arrows. The red dashed line is the new partition which divide clearly the maps. However, as shown in Supplementary Figure 4d-f, the embedded symbols with the partition always generate only periodic sequences of [A,B] even in the incommensurate state. Please compare the symbolic dynamics with the dynamics generated in the main text (see Fig. 3d-f). This result shows that the new partition is not suitable to be used for generating meaningful bit sequences.

Supplementary Note 5. Tree diagrams

From bit sequences in the incommensurate cases, we obtain tree diagrams (Supplementary Figure 5). The result shows dependences of the moving probability on their history which is related to the grammar of the symbolic dynamics. If the probabilities do not depend on their history, the dynamics can be considered suitable for a Markov system.

For the trees, we use a finite length of bit sequences (~9,300), thus it is not easy to be assured whether the dynamics is suitable for the Markov system or not. In the case of $I_{dc} = 13.0$ mA and 13.2 mA, all probabilities does not vary largely like one step memory dynamics. However, when $I_{dc} = 13.4$ mA, $P_{0,0}$ is

varied from 0.05 to 0.24 by the history. This change of the probability may originate from the dependence on the previous steps or may come from a lack of a number of the samples, which can increase the error of the probability.

In the main text, we assume one step memory dynamics, and obtained the probabilities, $P_{i,j}$ based on the Markov system. We also represent Markov matrices for each I_{dc} which corresponds with Fig. 4d in the main text.

Supplementary Note 6. Entropy rate of the Markov-Chain description of NCVO

According to Information Theory (See Ref. [3]), the entropy rate of a source $\{X_i\}_{i=1,n}$ is defined by

$$h_\infty = \lim_{n\to\infty} \frac{1}{n} H(X_1, \ldots, X_n) = \lim_{n\to\infty} H(X_n|X_{n-1}, \ldots, X_1). \tag{S3}$$

For a memoryless source, the conditional entropy satisfies $H(X_n|X_{n-1}, \ldots, X_1) = H(X_n)$ and as a result the entropy rate is equal to the binary entropy: $h_\infty = -P_0 \log_2(P_0) - (1 - P_0)\log_2(1 - P_0)$, which is maximum at 1 bit/symbol, if $P_0 = P_1 = 0.5$. Considering the Markovian property, we have $P(X_n|X_{n-1}, \ldots, X_1) = P(X_n|X_{n-1})$, thus simplifying the mathematical definition of the entropy rate $h_\infty = \lim_{n\to\infty} H(X_n|X_{n-1})$, and as a result, we can exploit the transition probability in the computation of the entropy rate. The binary symbols generated by the NCVO can be described using a binary Markov-chain representation with the following transition matrix

$$\pi = \begin{pmatrix} P_{0,0} & P_{0,1} \\ P_{1,0} & P_{1,1} \end{pmatrix} = \begin{pmatrix} 1-\alpha & \beta \\ \alpha & 1-\beta \end{pmatrix}. \tag{S4}$$

The stationnary probability distribution is given here by $\mu : \left[\mu_0 = \frac{\alpha}{\alpha+\beta}; \mu_1 = \frac{\beta}{\alpha+\beta}\right]$. The entropy rate then reads here $h_\infty = -\sum_{i,j=0,1} \mu_i P_{i,j} \log_2 P_{i,j}$, which simplifies into the following expression:

$$h_\infty = \frac{\beta}{\alpha+\beta} H_b(\alpha) + \frac{\alpha}{\alpha+\beta} H_b(\beta). \tag{S5}$$

Where the Shannon block entropy and the LZ complexity are maximum (at $I_{\mathrm{dc}} = 13.1$ mA), we have for the transition probabilities $\alpha = 0.4985$ and $\beta = 0.7430$. This leads to entropy rate for the NCVO:

$$h_\infty \approx 0.93 \text{ bit per symbol}. \tag{S6}$$

The analysis in terms of entropy rate of the Markov-chain description for the NCVO capture the asymptotic behaviour of the bit extracted from the NCVO chaotic alternation of patterns and shows that despite the asymmetry in transition probability, high level of entropy per symbol are achieved. This level

is consistent with the correlation analysis, with a lower bound for the KS entropy at $h_{\mathrm{KS}} \approx 0.12\ \mathrm{ns}^{-1}$ for $h_\infty \approx 0.093$ bit ns^{-1} (NB: on average, one binary symbol is emitted approximately every 10 ns by the NCVO, so the entropy rate h_∞ can be expressed in bit ns^{-1}).

Supplementary Note 7. Pattern recognition

In Figs. 3d-f in the main text, we show generated bit sequences from filtered time series, however, the data filtering takes substantial time. Here we discuss a method to directly extract the bits from the non-filtered data by using kernel functions. The process is described below:

1. Prepare kernel functions for 0 and 1, k_0 and k_1, (Supplementary Figure 6a) from preliminary measurements.

2. Measure a new time trace (Supplementary Figure 6b), then calculate the convolutions, c_0 (for k_0) and c_1 (for k_1), between the non-filtered data and the kernels as a function of shifting time (Supplementary Figure 6c).

3. Find the peaks from c_0 and c_1 whose heights are higher than tolerance values (circles and cross marks in Supplementary Figure 6c). Record the corresponding bit when the peaks appear (Supplementary Figure 6d).

We discard spurious peaks in c_1 which are very close to the peak positions in c_0. The cross marks in the bottom panel of Supplementary Figure 6c indicate the spurious peaks. We do not use these peaks for recording "1". In Supplementary Figure 6e, we plot the bit sequence obtained using the convolutions (red dots and curve), which is in a good agreement with that obtained from the filtered data (black squares and lines).

SUPPLEMENTARY REFERENCES

[1] Vansteenkiste, A. *et al.* The design and verification of MuMax3. *AIP Advances* **4**, 107133 (2014).

[2] Petit-Watelot, S., Otxoa, R. M. & Manfrini, M. Electrical properties of magnetic nanocontact devices computed using finite-element simulations. *Applied Physics Letters* **100**, 083507 (2012).

[3] Cover, T. M. & Thomas, J. A. *Elements of Information Theory* (Wiley, 2006), 2nd edn.
